# Supplementary material for: Nutritional Trade-Offs in Drosophila melanogaster
Source: Biology (Basel). 2025 Apr 7;14(4):384. doi: 10.3390/biology14040384 (PMC12024976; doi:10.3390/biology14040384)
Supplement: Supplementary file 1 [file biology-14-00384-s001.zip › biology-3473316-supplementary/FigureS1.pdf]

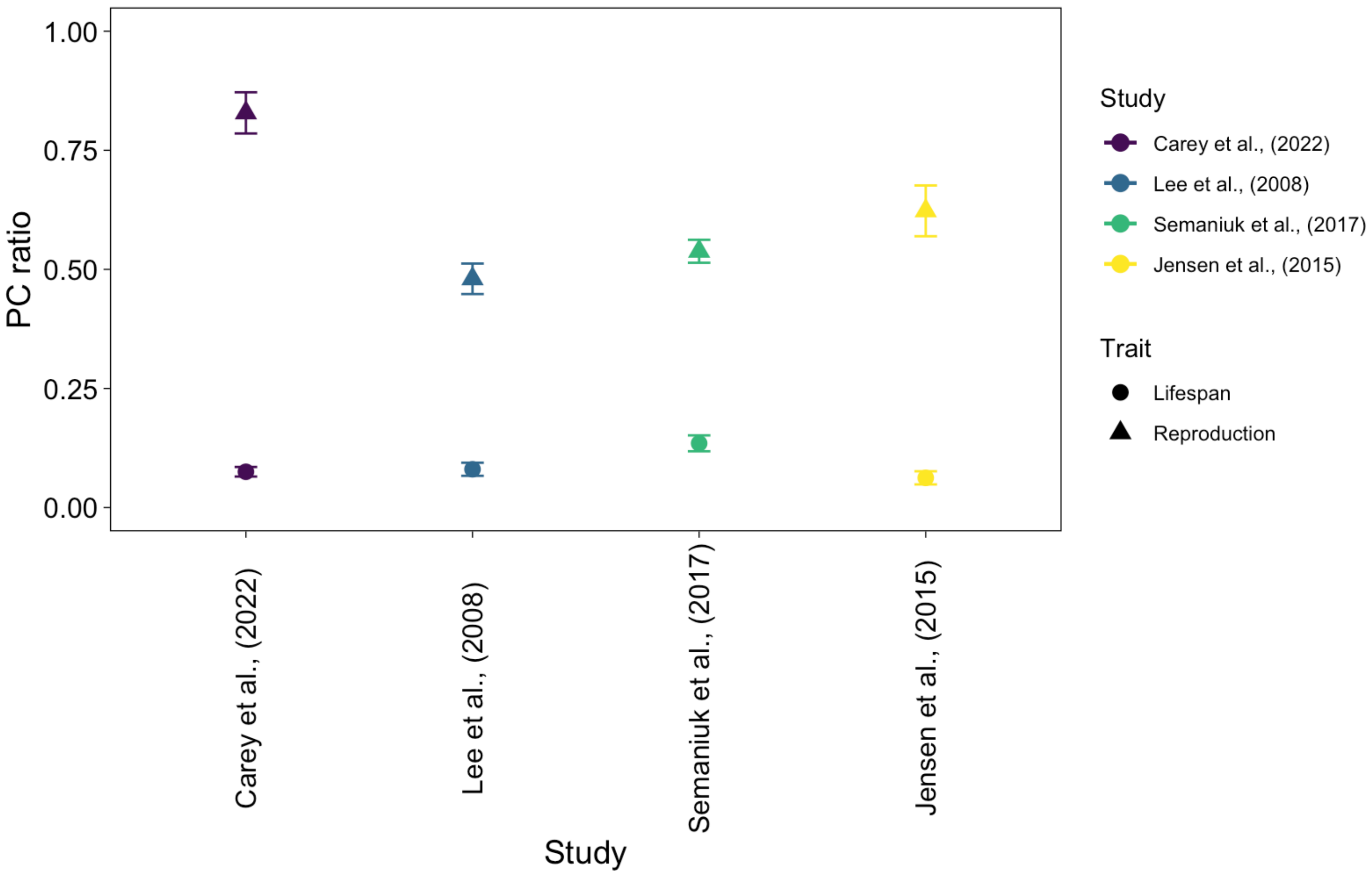

Figure S1. Peaks for lifespan (circle) and reproduction (triangles) across studies, highlighting the remarkable qualitative consistency of effect sizes across studies varying in methodology and genetic strains [6,19,30,53].
